# Hospitalist perspectives and practices in treating hospitalized patients with alcohol withdrawal syndrome in the era of phenobarbital re-emergence

**DOI:** 10.1002/jhm.70499

**Published:** 2026-10-04

**Authors:** James C. Willey, Julia E. Szymczak, Andrew D. Auerbach, Jeffrey L. Schnipper, Sunil Kripalani, Troy E. Madsen, Evan Done, Michael A. Incze, Valerie M. Vaughn, James D. Harrison

**Affiliations:** 1 Department of Internal Medicine, Division of General Internal Medicine, University of Utah, Salt Lake City, Utah, USA; 2 Department of Internal Medicine, Division of Epidemiology, University of Utah, Salt Lake City, Utah, USA; 3 Division of Hospital Medicine, University of California San Francisco, San Francisco, California, USA; 4 Harvard Medical School, Boston, Massachusetts, USA; 5 Hospital Medicine Unit, Division of General Internal Medicine and Primary Care, Brigham and Women’s Hospital, Boston, Massachusetts, USA; 6 Section of Hospital Medicine, Division of General Internal Medicine and Public Health, Vanderbilt University Medical Center, Nashville, Tennessee, USA; 7 Center for Health Services Research, Vanderbilt University Medical Center, Nashville, Tennessee, USA; 8 Department of Emergency Medicine, University of Utah, Salt Lake City, Utah, USA; 9 Department of Emergency Medicine, Intermountain Health Park City Hospital, Park City, Utah, USA; 10 Utah Support Advocates for Recovery Awareness, Salt Lake City, Utah, USA

## Abstract

Medication treatment for moderate to severe alcohol withdrawal syndrome (AWS) is evolving, particularly with renewed phenobarbital use. However, little research has examined how hospitalists manage AWS. We conducted a multiple-methods study involving hospitalists from a national US Hospital Medicine research network. Thirty-three hospitalists from 27 tertiary hospitals completed surveys and participated in seven focus groups on August 8, 2025. Benzodiazepines remained the most common treatment, but phenobarbital use was reported in 67% of emergency departments and 44% of inpatient medical units. Four themes emerged: hospitalists are adaptable in responding to changing practices and drug shortages; a lack of consensus on AWS treatment practices leads to a “wild west approach” to therapy selection; hospitalists prioritize care-delivery outcomes when selecting medications; and patients are minimally involved in shared decision-making. Participants strongly supported additional research to guide evidence-based AWS care. These findings highlight uncertainty and the need for standardized, patient-centered approaches to AWS management.

## INTRODUCTION

Hospitalization for alcohol withdrawal syndrome (AWS) is common, accounting for roughly 2 million admissions annually in the United States.^1,2^ AWS is a medical emergency requiring immediate attention and can lead to high resource utilization in hospitals, including multiple nursing assessments and long lengths of stay.^3^ Symptom-triggered dosing of benzodiazepines is the first-line, guideline-recommended treatment for moderate to severe AWS, but many providers are adopting phenobarbital either at a service level (e.g., in Emergency Departments [EDs]) or hospital-wide. ^3,4^ To date, there has been little research on national practice patterns in the use of phenobarbital or phenobarbital uptake in the United States.^5^ Most research has focused on the use of phenobarbital at single sites and, until recently, has primarily examined EDs and intensive care unit (ICU) settings.^6–8^ Phenobarbital has traditionally been reserved for severe benzodiazepine-refractory withdrawal, but it is increasingly being used to treat mild to moderate AWS or as the only therapy in a hospital.^9^ Phenobarbital’s reemergence as a primary AWS therapy has been driven by its pharmacokinetics (a single dose provides rapid AWS treatment), its mechanism of action (which is thought to be superior to benzodiazepines) and by benzodiazepine shortages.^8^ As AWS management evolves, there is a need to identify current hospitalist practices and describe ongoing challenges in AWS treatment protocols. Thus, we aimed to engage a national sample of hospitalists and describe: ED and inpatient medical unit AWS practices; hospitalists’ perspectives on current AWS practices and challenges with phenobarbital; and AWS outcomes that are important to hospitalists.

## METHODS

### Study design and oversight

We used a multiple-methods study design, including a 10-question survey and 7 virtual focus groups, to understand AWS practices and treatment challenges. The University of California San Francisco IRB exempted the study (#22-36424). We followed consolidated criteria for reporting qualitative research guidelines (see Supporting Information S1: Appendix A).^10^

### Context and setting

This study was conducted within the Hospital Medicine Reengineering Network (HOMERuN), a national consortium of academic hospital medicine programs dedicated to improving hospital care.^11^ The study surveys and small group interviews took place on August 8, 2025.

### Participants and sampling strategy

We used convenience sampling to recruit hospitalists from 50 HOMERuN sites via email for a virtual focus group on AWS treatment for hospitalized adults.

### Data collection

The study team created a survey and a focus group guide, with content informed by a combination of current approaches to AWS treatment, clinical expertise, and the study’s objectives.^12^ The 10-item survey, administered via Research Electronic Data Capture software, collected quantitative data from participants to assess ED and hospitalist treatment strategies for moderate-to-severe AWS (see Supporting Information S1: Appendix B). Seven focus group discussions were then completed that were guided by open-ended questions exploring participants’ perspectives on the current evidence for benzodiazepine and phenobarbital for treating AWS, how patient characteristics impact treatment decision-making, views on the goals/outcomes of AWS treatment, and patient involvement in treatment decision-making (see Supporting Information S1: Appendix C). Follow-up probes were used as needed to capture additional detail by allowing participants to clarify, elaborate, illustrate, or explain their responses.^13^ All focus group participants then took part in a report out to summarize their group’s discussion and to confirm the accuracy of data presented. Focus groups were digitally recorded, transcribed by an artificial intelligence transcription tool, and then checked for accuracy by J. D. H. Members of our research team (J. W., J. E. S., A. A., J. S., S. K., V. V., J. D. H.) served as moderators of the focus groups, following the developed guide. In some instances, the research team may have had professional knowledge or previous working relationships with the participants given the national networking that occurs in professional societies such as the Society of Hospital Medicine (SHM) and HOMERuN.

### Analysis

Survey data were summarized with descriptive statistics. Hospital-specific responses from hospitalists practicing together were collapsed. Focus group data were analyzed using reflexive thematic analysis.^14^ Transcripts were analyzed by two members of the research team (J. W., J. D. H.), who have experience in qualitative research, for accuracy and initially read twice to familiarize the researchers with the data. Then, transcripts were coded via provisional, inductive (data-driven) line-by-line coding to describe the entire data set. We held team meetings to establish consensus regarding the meaning of text and codes. Data relevant to each code were iteratively summarized and re-arranged following the establishment of team consensus. Codes and data were then grouped into potential themes related to the practices and challenges of providing AWS care. Qualitative data analysis was managed using Microsoft Excel (Version 16.111.1). We took several steps to ensure theoretical and methodological rigor and credibility throughout the analysis.^15,16^ These included sustained immersion and familiarization with the data, independent coding, group meetings to review the data and reach coding consensus, maintenance of an electronic paper trail of analytic decisions, and member checking with multiple participants.

## RESULTS

Of the 50 hospital sites invited, 27 hospital sites (54%) participated in this study, completed the survey, and took part in focus groups. The 33 hospitalists participating were from hospitals across the Northeast (7), Midwest (6), South (8), and West (6) census districts. Phenobarbital was reportedly used to treat AWS in 67% (18) of EDs and 44% (12) of acute medical units; phenobarbital was the only AWS treatment in 30% (8) of EDs and 7% (2) of acute medical units (Table 1). Most hospital sites used Clinical Institute Withdrawal Assessment for Alcohol-Revised (CIWA-AR) scores (74%) to assess AWS severity and used lorazepam (67%) as the benzodiazepine of choice for AWS treatment.

Focus group analysis identified four themes, each with subthemes (see Appendix D for coding Tree). The first theme highlighted how hospitalists are adaptable and capable of providing AWS care as they concurrently adjust to changing practice patterns and drug shortages. Benzodiazepines remain the primary medication used by hospitalists on the floor, but increasingly, phenobarbital is being introduced. The second theme centered on concerns that the lack of evidence around some AWS treatments led to variability in treatment practices within hospitals (e.g., between providers and units). This practice variation was exacerbated in part by hospital policies regarding where phenobarbital can be administered. The third theme described how hospitalists prioritized AWS outcomes. Specifically, hospitalists felt that the most important and challenging factor driving AWS treatment decisions was efficient use of hospital resources (nursing time, ICU beds) rather than effective treatment of withdrawal. Institutions that primarily used phenobarbital believed it was more effective in treating hospitalized AWS, required less nursing care, provided more consistent withdrawal control, and increased patient satisfaction—although these beliefs were based on clinical impressions rather than data. The final theme highlighted a lack of patient involvement in shared decision-making and informed consent for AWS treatment. A repeated unifying comment across themes was that hospitalists felt more research was needed to answer the question of best therapy for AWS, including studies that compared phenobarbital and benzodiazepine therapies. Table 2 describes themes, subthemes, and representative quotes. Supporting Information S1: Appendix E provides additional participant quotes for each theme/subtheme.

## DISCUSSION

In our multiple-methods study, we found phenobarbital treatment of AWS is common in EDs and acute medical units, though only a minority of hospitals reported using phenobarbital exclusively. Focus groups raised concerns about the variability in AWS treatment practices within a hospital (e.g., between providers and service levels). Hospitalists consistently reported that more research was needed to determine the best therapy for AWS. Currently, one study at a single site evaluates a hospital-wide phenobarbital order set, but no trials on clinicaltrials.gov compare phenobarbital to benzodiazepines for AWS.^8^

Our study expands our understanding by providing a national perspective on hospitalists’ current practices and their adoption of phenobarbital pharmacotherapy for AWS. We found that over half of EDs were using phenobarbital, while fewer than half of the acute medical floors were. While phenobarbital use is rising on the floors, it is still more common in the EDs, which often leads to patients starting on phenobarbital in the ED and then switched to benzodiazepine therapy on the floor (due to hospital policy restricting phenobarbital use on the floor).

Hospitalists voiced confidence in managing withdrawal and adapting to changing practices, drug and drug shortages, but uniformly felt they needed more evidence to guide practice on acute medical floor treatment of AWS. Even hospitalists using phenobarbital feel further support is required (Supporting Information S4: Appendix E Theme 2 subtheme a). Treatment decisions often rely on provider preference, comfort, or protocols rather than data, risking inconsistent care, higher toxicities, and medication interactions with combined phenobarbital and benzodiazepines.

In addition to the recurrent voiced need for further research to establish practice standards across hospitals, hospitalists felt that the major challenge for AWS treatment was resource utilization, rather than withdrawal control. Hospitalists were interested in comparing benzodiazepines and phenobarbital in terms of the need for nursing intervention, ICU time required, and length of stay, followed by patient symptom control and decreased complications of withdrawal. This emphasis may reflect both the high level of resources needed for this population, pressures on hospitalists from hospital administration, and bias against those with alcohol use disorder.^17^

Focus group discussions revealed challenges in shared decision-making when patients are intoxicated or severely withdrawing. Most hospitalists reported not engaging in shared decision-making for inpatient AWS, with hospital restrictions on medications also limiting options.

Our study has several strengths, including a multiple-methods approach, the inclusion of a range of hospitalists, and representation from multiple academic medical centers. However, some study limitations include a 54% participation rate of HOMERuN hospital sites, and a reliance on self-reports from hospitalists interested in AWS treatment who are mainly from academic or tertiary medical centers. These factors may limit the generalizability of findings and reaching thematic saturation. While focus group participants took part in a discussion to check the accuracy of data, we did not complete member checking on the finalized set of themes. Finally, this study captures only hospitalists’ perspectives and excludes other clinicians. This study lays a foundation for future research by highlighting clinician equipoise, the need for more data, and by suggesting that resource utilization is a major factor in choosing between effective withdrawal treatments.

## CONCLUSION

Hospitalists reported that benzodiazepines were the most common AWS treatment on the acute medical floor, but phenobarbital was increasingly available and used in the ED. Hospitalists were concerned about the lack of evidence to guide AWS treatment and the high variation in treatment approaches within and across hospitals. In response, hospitalists strongly advocated research on inpatient AWS treatment protocols to guide evidence-based practice and support a uniform approach to AWS.

## Supplementary Material

Supplementary Material A - COREQ (COnsolidated criteria for REporting Qualitative research) Checklist

Supplementary Material B - Clinician Survey

Supplementary Material C - Moderator Protocol

Supplementary Material D - Coding Tree

Supplementary Material E - Additional participant quotes for each theme/subtheme.

Additional supporting information can be found online in the Supporting Information section at the end of this article.

## Figures and Tables

**TABLE 1:** Current AWS treatment practices and AWS order set use (*n* = 27 hospitals).

Phenobarbital and benzodiazepine use		
	Emergency department, *n* (%)	Medical floor, *n* (%)
Pb as the only treatment for AWS	8 (30)	2 (7)
Any Pb use	18 (67)	12 (44)
High initial dose Pb^a^	12 (44)	7 (26)
Low initial dose Pb^b^	12 (44)	11 (41)
Any BZD use	19 (70)	25 (93)
BZD front load^c^	11 (44)	10 (37)
BZD STT^d^	18 (67)	23 (85)
Pb load then BZD STT	1 (4)	4 (15)

Scale used for symptom-triggered therapy, *n* (%)		
CIWA-Ar	20 (74)	
MINDS	2 (7)	
BAWS	1 (4)	
RASS (On acute medical floors)	2 (7)	
RASS (ICU only)	5 (19)	
AWAS	1 (4)	
No answer	1 (4)	

Primary benzodiazepine used for alcohol withdrawal syndrome, *n* (%)		
Lorazepam	18 (67)	
Diazepam	9 (33)	

*Note*: Pb Load: 10 mg/kg or more initial IV bolus of phenobarbital.

Abbreviations: AWAS, Alcohol Withdrawal Assessment Scale; AWS, alcohol withdrawal syndrome; BAWS, Brief Alcohol Withdrawal Scale; BZD, benzodiazepine; CIWA-Ar, Clinical Institute Withdrawal Assessment for Alcohol-Revised; ED, emergency department; ICU, intensive care unit; MINDS, Minnesota Detoxification Scale; Pb, phenobarbital; RASS, Richmond Agitation-Sedation Scale; Rx, medical prescription; STT, symptom-triggered therapy.

a High initial Pb dose: initial IV bolus dose of 10-15 mg phenobarbital per kg of the patient’s ideal body weight, followed by symptom-triggered dosing.

b Low initial Pb dose: 65–260 mg initial IV bolus followed by symptom-triggered dosing.

c BZD front load: Any fixed dose benzodiazepine protocol at the start of AWS or any benzodiazepine protocol with doses varying on severity of AWS scale.

d BZD STT: benzodiazepine symptom-triggered therapy.

**TABLE 2:** Focus group themes and sub-themes: Hospitalist perspectives on AWS treatment, practice, and research needs.

**Theme 1: Hospitalists are adaptable and capable of providing AWS care as they adjust to changing practice patterns and drug shortages**
**Theme 1 sub-theme a: Benzodiazepines are the primary medication used by hospitalists on the floor for AWS** *We [hospitalists] have an EPIC order set for both mild to moderate and moderate to severe, which is based in using Lorazepam*. *We admit the patients and then immediately we start them on the benzo protocol. We don’t have a phenobarb on the floor protocol, but if the symptoms are worse, if the score, if they really score high and the benzos are not helping, they get the second [phenobarbital] dose*.
**Theme 1 subtheme b: Phenobarbital use for AWS is increasing on floors, and hospitalists want the option to use it** *We utilize a phenobarbital protocol across all our hospitals. It’s been in place for four or five years*. *We recently started a phenobarbital order set, so it’s still new for us and in fact we’re still doing education for our hospitalists as well as for nursing*.
**Theme 1 subtheme c: Treatment choices are influenced by medication shortages** *I think for us, honestly, it was that Ativan shortage that really stimulated that conversation about phenobarbital* *I was on a call this morning about our remaining supply of Lorazepam. So we are looking for opportunities to utilize other agents and there’s kind of a shift that we’re all experiencing and wanting to use more phenobarbital*
**Theme 2: A “wild west” or “vibes-based approach” to AWS therapy selection**
**Theme 2 sub-theme a: Wild West – a lack of evidence or rules to guide therapy selection for inpatient medicine** *[AWS Treatment]…is a bit of a mishmash right now and so the confusion is a problem. The nurses don’t know what to do, the residents don’t know what to do — it’s basically the wild west again* *We recognize that there’s not a lot of literature (on phenobarbital AWS treatment in acute medical floors)…Mostly we’re borrowing from the ICU or the ED and they seem to be very comfortable with it, but it makes us (hospitalists) nervous*…
**Theme 2 subtheme b: Vibes-based approach, providers felt care was determined by the unit or provider preference** *I feel like this research and this data is really important to have because it just seems to be very stylistic. At least what I’ve seen, not everybody, even in our psych department, is bought in on this [treatment with phenobarbital]*. *One thing I’ve learned about alcohol withdrawal is everyone kind of just does what they were trained with. No one’s very comfortable switching*.
**Theme 3: Hospitalist felt that the most important factor driving AWS treatment decisions was efficient use of hospital resources (nursing time, ICU beds) rather than effective treatment of withdrawal**
**Theme 3 subtheme a: Hospital factors: Nursing burdens, length of stay and ICU utilization** *Nurse survey questions on ease of care of these patients would be really interesting because as much as we struggle at times with these patients, it’s the nurses on the front line that have the real brunt of it. Some nurse experience data would be really interesting to follow if there’s any difference, maybe there isn’t. It would be interesting to see*. *Length of stay from the hospital perspective…also any kind of clinical deterioration or ICU*
**Theme 3 subtheme b: Patient safety** *I think if we could show that there was a decrease in escalations, decreased use of restraints, decreased incidents of behavioral issues, then I think more people would be on board with trying to use phenobarbital* *The safety outcomes (respiratory depression, hypotension oversedation, encephalopathy, delirium, ICU escalation) are really important because I think what happens with … benzodiazepines in the setting of alcohol withdrawal is they’re given a free pass kind of*.
**Theme 4: No shared decision-making for treatment of AWS.**
**Theme 4 subtheme a: Acute intoxication or alcohol withdrawal limits decision-making** *If the patient is already in severe alcohol withdrawal, then sometimes the discussion just cannot be done because of the mental status of the patient*. *I don’t see them being too to engaged in choosing one therapy versus another. A lot of ‘em might be completely disoriented, but unless they’ve had an allergy or a bad response to a medication previously, I don’t think they’re going to object to one versus another*.
**Theme 4 subtheme b: AWS treatment selection is determined by hospital protocol** *It is our default EPIC protocol …. this is what you’re getting. I think we’re comfortable telling people this is our protocol. We have a high level of comfort with this, a high level of success rate with this*. *Because we don’t have a written protocol for phenobarb, we’ve never been able to say, okay, we’ll switch. I think if we had both protocols available in the order set, if a patient asked to switch it, then would be easy. But right now it’s not available to us*.
**Theme 4 subtheme c: Patients are not given an option to decide AWS treatment** *Hospitalist 1: I would say in my 20 years of experience as a hospitalist, there’s no engagement in these patients, whether they prefer one medication or another*. *Hospitalist 2: Yep. I second that also wth 20 plus years experience*.

## Data Availability

The data that support the findings of this study are available from the corresponding author upon reasonable request.
