# Supplementary material for: Hospitalist perspectives and practices in treating hospitalized patients with alcohol withdrawal syndrome in the era of phenobarbital re-emergence
Source: J Hosp Med. Author manuscript; Available in PMC 2026 Oct 23. (PMC13693485; doi:10.1002/jhm.70499)
Supplement: Supplementary file 2 [file NIHMS2214780-supplement-Supplementary_Material_B_-_Clinician_Survey.pdf]

HOMERuN PCORI AWARD 8-8-2025 survey

Your name: ________________________________

Primary hospital you work with (if you work at more than one, please answer the following questions for only one hospital): ________________________________

Email:
If you would like us to contact you about participating in this study. ________________________________

Which of the following strategies does your hospital currently use to treat patients with moderate to severe alcohol withdrawal in the emergency department? (select all that apply)

- ☐ Phenobarbital, low initial dose (e.g., 65-260 mg initial IV bolus followed by symptom-triggered dosing)
- ☐ Phenobarbital, front-loaded (e.g., 10-15 mg/kg initial IV bolus followed by symptom-triggered dosing)
- ☐ Benzodiazepine, front loaded (i.e., scheduled intervals of BZD dosing and taper)
- ☐ Benzodiazepine, symptom triggered (i.e., PO/IV BZD only triggered by symptom score)
- ☐ Other

Please briefly describe: ____

Which of the following strategies does your hospital currently use to treat patients with moderate to severe alcohol withdrawal in the hospital wards (floor)? (select all that apply)

- ☐ Phenobarbital, low initial dose (e.g., 65-260 mg initial IV bolus followed by symptom-triggered dosing)
- ☐ Phenobarbital, front-loaded (e.g., 10-15 mg/kg initial IV bolus followed by symptom-triggered dosing)
- ☐ Benzodiazepine, front loaded (i.e., scheduled intervals of BZD dosing and taper)
- ☐ Benzodiazepine, symptom triggered (i.e., PO/IV BZD only triggered by symptom score)
- ☐ Other

Please briefly describe: ____

What is the most commonly used benzodiazepine for patients with active alcohol withdrawal admitted to the inpatient (floor) service at your hospital?

- ◯ Librium (Chlordiazepoxide)
- ◯ Ativan (lorazepam)
- ◯ Valium (diazepam)
- ◯ Other

Please briefly describe: ________________________________

Does your hospital have an inpatient addiction medicine/psychiatry service?

- ◯ No
- ◯ Yes, but they do NOT see patients with alcohol use disorder
- ◯ Yes, but only when asked (i.e., medical team orders a consult)
- ◯ Yes, for all patients with alcohol withdrawal (i.e., automatic consult)

Who writes the inpatient orders for treatment of alcohol withdrawal?	◯ Primary medical team<br>◯ Addiction medicine/psychiatry service
Does your hospital have a detox facility where you send most of your patients with alcohol withdrawal? (i.e., patients requiring medical treatment for alcohol withdrawal)	◯ Yes<br>◯ No<br>◯ Other
Please describe:	__________________________________
Does your hospital use a symptom triggered scale for alcohol withdrawal? (e.g., CIWA)	◯ Yes<br>◯ No
Which type:	☐ CIWA-Ar<br>☐ BAWS<br>☐ MINDS<br>☐ RAAS (outside of the ICU or ED intubated patients)<br>☐ RAAS (ICU only)<br>☐ Other
Please describe:	__________________________________
Do you think hospitalists at your hospital would be willing to be part of a study that randomizes patients with active alcohol withdrawal to receive either phenobarbital vs. benzodiazepines?	◯ Yes<br>◯ Maybe<br>◯ No<br>◯ Unsure
Please explain:	__________________________________
