# Supplementary material for: Hospitalist perspectives and practices in treating hospitalized patients with alcohol withdrawal syndrome in the era of phenobarbital re-emergence
Source: J Hosp Med. Author manuscript; Available in PMC 2026 Oct 23. (PMC13693485; doi:10.1002/jhm.70499)
Supplement: Supplementary file 3 [file NIHMS2214780-supplement-Supplementary_Material_C_-_Moderator_Protocol.docx]

**Appendix C: Moderator Protocol**

**AWARD Trial HOMERuN Focus Group Questions 8-8-2025**

**25-30 minutes/10 minutes report out**

**Remember to:**

1. Hit RECORD
2. Enter survey link in chat: {HYPERLINK}
3. **Start discussion by** having everyone give their name, role (e.g., hospitalist), and describe the current alcohol withdrawal treatment protocol at their hospital.
4. Send in in the chat toward the end: Please put your name/site in the chat if you’re interested in being a clinical trial site or a dissemination site.

**Questions for discussion:**

1) How important do you think this research is to hospital practice? (i.e., comparing BZD to PB and different strategies within each)

Notes:

2) Are there any clinical characteristics that you feel need to be taken into account when choosing between these treatments? (Could be made into subgroups, exclusions, treatment variations)

Prompt: example (if needed) BZD resistance would be a factor in treatment selection

Notes:

3) **What outcomes would you like to see evaluated in a trial of these different medications?** What evidence would you need to see to change your approach to medication use in these patients?

Notes:

4) How often are patients engaged in shared decision-making in deciding what medications and what treatment strategy to use for alcohol withdrawal management while they are hospitalized? What are potential barriers to this type of shared decision-making?

Notes:

5) What concerns (if any) do you have about any of the treatment arms?

Notes:

**After focus group:**

Add to chat at the end, please put your name/site in the chat if you’re interested in being a clinical trial site or a dissemination site.
