# Supplementary material for: Hospitalist perspectives and practices in treating hospitalized patients with alcohol withdrawal syndrome in the era of phenobarbital re-emergence
Source: J Hosp Med. Author manuscript; Available in PMC 2026 Oct 23. (PMC13693485; doi:10.1002/jhm.70499)
Supplement: Supplementary file 5 [file NIHMS2214780-supplement-Supplementary_Material_E_-_Additional_participant_quotes_for_each_theme_subtheme_.docx]

**Appendix E:**

**Theme 1: Hospitalists are adaptable and capable of providing AWS care as they adjust to changing practice patterns and drug shortages**

**Theme 1 sub-theme a: Benzodiazepines are the primary medication used by hospitalists on the floor for AWS**

We mostly use benzos on the floors

The general medicine service uses CIWA and it is nursing driven, mostly Lorazepam.

[The ED will provided high doses of phenobarbital in moderate to severe AWS]… then we [hospitalist] have an EPIC order set for both mild to moderate and moderate to severe, which is based in using Lorazepam.

[hospitalist] We’re using a lot of reflexive benzodiazepines with mostly Lorazepam, which is on shortage, but we're using that as the backbone of our alcohol draw protocol at this moment

[all ED patients with AWS are loaded with phenobarbital] we admit the patients and then immediately we start them on the benzo protocol. We don't have a phenobarb on the floor protocol, but if the symptoms are worse, if the score, if they really score high and the benzos are not helping, they get the second [phenobarbital] dose.

I would say we [hospitalists] have mostly been using just Benzo. However, I just collected all the evidence, gave grand rounds, and now we are switching to phenobarbital.

We [hospitalists] currently use a Valium-based protocol based on CIWAs that is nursing-driven.

Most people are doing symptom-triggered benzo with Ativan

The ER and the medicine floors use CIWA symptom-triggered protocol with either Librium or Valium, occasionally Ativan. Unfortunately, phenobarbital is currently restricted to the ICU

The ED uses Valium or Ativan. On the floors, we use either standing or PRN [benzodiazepines], depending on the patient. And we just got a nurse-driven CIWA protocol approved

In the ED, if the patient has had a historically severe withdrawal, they will do a phenobarbital front load and then once they hit the floor, it's a benzodiazepine-based symptom-triggered protocol.

The default order set is, uh, lorazepam triggered by CIWA.

So focusing on using things like, you know, high dose gabapentin, clonidine, and then using, um, CIWA triggered benzodiazepine on top of that

**Theme 1 sub-theme b:** **Phenobarbital use for AWS is increasing on floors, and hospitalists want the option to use it**

We [hospitalist] are already thinking about an intravenous front-load phenobarbital protocol

We utilize a phenobarbital protocol across all our hospitals. It's been in place for by four or five years.

The emergency department or the admitting hospitalist chooses to continue either phenobarbital or Lorazepam using either a standard or reduced symptom-based dosing regimen.

We recently started a phenobarbital order set, so it's still new for us and in fact we're still doing education for our hospitalists as well as for nursing.

(regarding acute medical floor treatment of AWS) I think our less complicated patients are still getting CIWA scored Ativan, and our more complicated patients, where we used to do a lot of fixed Ativan tapers, we're now doing a lot more phenobarb

Once a patient moves to hospital medicine, we vary between using STT-driven benzodiazepine therapy versus the phenobarbital protocol

We front-load them either with IV Diazepam or IV phenobarb, depending on the patient

**Theme 1 sub-theme c: Treatment choices are influenced by medication shortages**

Because of the shortage, we are now using diazepam when Ativan is not readily available

I think for us, honestly, it was that Ativan shortage that really stimulated that conversation about phenobarbital, didn't necessarily do anything else. And I'm seeing nods from everybody else

I'd say probably once every two years or so [there is a benzodiazepine shortage] has been my experience. We tend to use Lorazepam, but we've had shortages of just about everything at various times.

I think the only reason we started using phenobarb again was that there was a shortage of IV benzos for a while and then there came out some evidence showing that patients could be treated with phenobarbital.

Part of it was because of Ativan shortage and they kind of wanted to see the alternative pathway

Because of the shortage we are now using diazepam when Ativan is not readily available.

I was on a call this morning about our remaining supply of Lorazepam. So we are looking for opportunities to utilize other agents and there's kind of a shift that we're all experiencing and wanting to use more phenobarbital

**Theme 2: A “wild west” or “vibes-based approach” to AWS therapy selection**

**Theme 2 sub-theme a: Wild West – a lack of evidence or rules to guide therapy selection for inpatient medicine**

*[AWS Treatment]…is a bit of a mishmash right now and so the confusion is a problem. The nurses don’t know what to do, the residents don't know what to do — it's basically the wild west again*

*We recognize that there's not a lot of literature (on phenobarbital AWS treatment in acute medical floors)…Mostly we're borrowing from the ICU or the ED and they seem to be very comfortable with it, but it makes us (hospitalist) nervous, but I think it shouldn't theoretically.*

*We don't have any really high quality studies looking at phenobarb compared to benzos, especially long acting…and you can tell even just in this small focus group, everybody's doing different things.*

*I think our challenge has been the lack of data on floor patients…*

*Our [phenobarbital] experience has been very positive. Once again, it's always helpful to have actual randomized control trial data …[to support] why we do this.*

*It would be great to have more data …I think that's what we need because right now we just don't have really enough data to move fully to phenobarbital without potentially putting patients at risk. I think it's hard to move to something that could cause harm when we have so much data to support the use of benzos*

**Theme 2: sub-theme b: Vibes-based approach, providers felt care was determined by the unit or provider preference**

*Almost everybody is just started on phenobarbital [in the emergency department] and then it is just up to attending hospitalists to decide how to proceed. I would say we mostly have been using just benzos.*

*We're currently doing what I view to be maybe the worst of all the things where we're mixing them a lot, where the ED is kind of loading either small dose or large dose depending on the individual provider.*

*In the ED, it's very provider dependent, whether to load one time with phenobarbital versus resort to benzodiazepines*

*I feel like this research and this data is really important to have because it just seems to be very stylistic. At least what I've seen, not everybody, even in our psych department, is bought in on this [treatment with phenobarbital]. So if you consult psych and it's not one of the right attendings[those who use phenobarbital], they'll be like, we don't consult this because we don't know how to give you advice on this [phenobarbital treatment of AWS]*

*One thing I've learned about alcohol withdrawal is everyone kind of just does what they were trained with. No one's very comfortable switching.*

**Theme 3: Hospitalist felt that the most important factor driving AWS treatment decisions was efficient use of hospital resources (nursing time, ICU beds) rather than effective treatment of withdrawal**

**Theme 3 sub-theme a: Hospital factors: Nursing burdens, length of stay and ICU utilization**

*Nurse survey questions on ease of care of these patients would be really interesting because as much as we struggle at times with these patients, it's the nurses on the front line that have the real brunt of it. Some nurse experience data would be really interesting to follow if there's any difference, maybe there isn't. It would be interesting to see.*

*Length of stay from the hospital perspective…also any kind of clinical deterioration or ICU*

**Theme 3 sub-theme b: Patient safety**

*Nurse survey questions on ease of care of these patients would be really interesting because as much as we struggle at times with these patients, it's the nurses on the front line that have the real brunt of it. Some nurse experience data would be really interesting to follow if there's any difference, maybe there isn't. It would be interesting to see.*

*Length of stay from the hospital perspective…also any kind of clinical deterioration or ICU*

*I think if we could show that there was a decrease in escalations, decreased use of restraints, decreased incidents of behavioral issues, then I think more people would be on board with trying to use phenobarbital*

*The safety outcomes (respiratory depression, hypotension oversedation, encephalopathy, delirium, ICU escalation), really important because I think what happens with what's happened with benzodiazepines in the setting of alcohol draw is they're given a free pass kind of.*

**Theme 4: No shared decision-making for treatment of AWS.**

**Theme 4 sub-theme a: Acute intoxication or alcohol withdrawal limits decision-making**

*If the patient is already in severe alcohol withdrawal, then sometimes the discussion just cannot be done because of the mental status of the patient.*

*I don't see them being too to engaged in choosing one therapy versus another. A lot of 'em might be completely disoriented, but unless they've had an allergy or a bad response to a medication previously, I don't think they're going to object to one versus another.*

**Theme 4 sub-theme b: AWS treatment selection is determined by hospital protocol**

*It is our default epic protocol …. this is what you're getting. I think we're comfortable telling people this is our protocol. We have a high level of comfort with this, a high level of success rate with this.*

*A lot of us really only have one way of doing things, so we're not really able to even present, um, options to them in a very, like, academic way.*

*Because we don't have a written protocol for phenobarb, we've never been able to say, okay, we'll switch. I think if we had both protocols available in the order set, if a patient asked to switch it, then would be easy. But right now it's not available to us.*

**Theme 4 sub-theme c: Patients are not given an option to decide AWS treatment**

***Hospitalist 1****: I would say in my 20 years of experience as a hospitalist, there's no engagement in these patients, whether they prefer one medication or another.*

***Hospitalist 2:*** *Yep. I second that also with 20 plus years experience,*

*I don't think I've ever asked a patient which medication they wanted to use for their withdrawal, to be totally honest with you.*
