# Supplementary material for: Hospitalist perspectives and practices in treating hospitalized patients with alcohol withdrawal syndrome in the era of phenobarbital re-emergence
Source: J Hosp Med. Author manuscript; Available in PMC 2026 Oct 23. (PMC13693485; doi:10.1002/jhm.70499)
Supplement: Supplementary file 6 [file NIHMS2214780-supplement-Supplementary_Material_D_-_Coding_Tree.docx]

Appendix D: Thematic Analysis Coding Tree
